# Cathether-based interventional strategies for cor triatriatum in the adult – feasibility study through a hybrid approach

**DOI:** 10.1186/s12872-015-0067-4

**Published:** 2015-07-14

**Authors:** Wilson W. Li, David R. Koolbergen, Berto J. Bouma, Mark G. Hazekamp, Bas A. de Mol, Robbert J. de Winter

**Affiliations:** Department of Cardiothoracic Surgery, Academic Medical Center, University of Amsterdam, Meibergdreef 9, 1105 AZ Amsterdam, The Netherlands; Department of Cardiology, Academic Medical Center, University of Amsterdam, Meibergdreef 9, 1105 AZ Amsterdam, The Netherlands

**Keywords:** Congenital heart disease, Cardiovascular intervention, Cardiac surgery

## Abstract

**Background:**

Cor triatriatum is a rare congenital cardiac abnormality, consisting of an obstructing membrane between the pulmonary veins and the mitral valve in varying patterns. The entitiy can mimick the pathophysiology of mitral stenosis, necessitating surgical resection. Occasionally, percutaneous balloon dilatation of the membrane has been successfully performed.

**Case presentation:**

We report two cases with cor triatriatum where intraoperative balloon dilatation of the membrane was attempted followed by surgical resection, to explore the feasibility of cathether-based interventional strategies for cor triatriatum.

**Conclusions:**

Various anatomical variations exist of cor triatriatum, depending on the drainage of the pulmonary veins and the drainage of the proximal chamber in the right or left atrium. Only isolated forms of cor triatriatum where all pulmonary veins ultimately drain into the left atrium can be recommended for percutaneous strategies. In addition, several anatomical characteristics should be considered to predict technical success of cathether-based interventional strategies, such as the location of the membrane, the degree of pulmonary vein stenosis, the extent of calcification, and the presence of other (congenital) cardiovascular abnormalities. Furthermore, long-term efficacy of these strategies remains to be confirmed. As such, surgical treatment of cor triatriatum remains the mainstay of treatment in adult patients, especially when other cardiovascular anomalies are present which require surgical correction.

## Background

Cor triatriatum is a rare congenital cardiac abnormality, with a reported incidence of <0.1–0.4 % in patients with congenital heart disease [1, 2]. Various anatomical variations exist and can be best categorized according to the modified classification of Lucas [3] (Table 1). The most common form, classic cor triatriatum, is characterized by the presence of a common pulmonary venous chamber (proximal or posterior chamber) which is separated from the left atrium (distal or anterior chamber) by a fibromuscular membrane [3]. The communication between these two chambers may vary in size. When this opening is significantly obstructive, it causes restriction of pulmonary venous return and possibly pulmonary hypertension, mimicking the pathophysiology of mitral stenosis. In these cases, surgical resection of the membrane is indicated, with excellent long-term outcome [4, 5].

**Table 1:** Modified classification of cor triatriatum according to Lucas [3]

I.	Accessory atrial chamber receives all pulmonary veins and communicates with left atrium
	A. No other connections (classic cor triatriatum)
	B. Other anomalous connections
	1. To right atrium directly
	2. With totally anomalous pulmonary venous connection
II.	Accessory atrial chamber receives all pulmonary veins and does not communicate with the left atrium
	A. Anomalous connection to right atrium directly (cardiac TAPVC with all pulmonary veins first draining to a venous confluence)
	B. With totally anomalous pulmonary venous connection (supracardiac or infracardiac TAPVC)
III.	Subtotal cor triatriatum
	A. Accessory atrial chamber receives part of the pulmonary veins and connects to the left atrium
	1. Remaining pulmonary veins connect normally
	2. Remaining pulmonary veins connect anomalously (partial cor triatriatum with PAPVC)
	B. Accessory atrial chamber receives part of the pulmonary veins and connects to the right atrium
	1. Remaining pulmonary veins connect normally (PAPVC with anomalously connected pulmonary veins first draining to a venous confluence)
	2. Remaining pulmonary veins connect anomalously (mixed TAPVC)

*PAPVC* Partially anomalous pulmonary venous connection, *TAPVC* Totally anomalous pulmonary venous connection

Reprint from Herlong et al. [3]

Advances in percutaneous cardiovascular interventions have allowed percutaneous treatment of cardiac diseases that previously required surgical therapy. For example, percutaneous mitral commissurotomy (PMC) has become the treatment of choice in symptomatic patients with significant mitral stenosis with favourable valve anatomy [6, 7]. Correspondingly, successful percutaneous balloon dilatation of cor triatriatum has also been reported occasionally. First by Kerkar [8] in 1996, and subsequently mainly in children [9–11] and young adults [8, 12, 13] (Table 2). Currently, there are no guidelines or recommendations regarding which of the anatomical variations are appropriate for percutaneous or surgical treatment.

**Table 2:** Overview of published reports on percutaneous interventions in cor triatriatum

Author	Journal	Age	Cor triatriatum classification	Technique	Outcome and remarks	Follow-up
Kerkar [8]	Am Heart J 1996	16 y	IA	Double-balloon dilatation (2× 18 mm diameter & 3 cm-long balloon angioplasty catheter sequentially placed)	Reduction of transmembrane gradient from 34 to 4 mmHg, and pulmonary artery pressure from 92/48 to 36/16 mmHg	3 m
Huang [9]	Catheter Cardiovasc Interv 2002	8 y	IA	Inoue balloon dilatation	Reduction of transmembrane gradient from 26 to 4 mmHg	12 m
Sivakumar [12]	Pediatr Cardiol 2008	30 y	IIIA1	Balloon dilatation (16 mm × 4 cm Tyshak II balloon)	Reduction of pressure in proximal chamber from 32 to 12 mmHg	3 m
Schiller [10]	Pediatr Cardiol 2012	3 m	IIIA2	Balloon dilatation (13 mm)	Admitted to emergency department with cardiogenic shock due to obstructing cor triatriatum and PAPVC. Planned staged treatment with percutaneous intervention as palliative measure before definitive surgical therapy.	9 m
					Reduction of transmembrane gradient from 20 to 1 mmHg	
Mendez [13]	European Journal of Heart Failure 2013	30 y	IA	Inoue balloon dilatation (30 mm)	Reduction of transmembrane gradient from 20 to 1 mmHg, and increase of orifice diameter from 1.2 to 2 cm	6 m
Schranz [11]	Catheter Cardiovasc Interv 2013	3 m	IIB	Placement of 7 × 16 mm stent	Complex congenital heart defect with HLHS, TAPVC, cor triatriatum. Percutaneous intervention as part of staged treatment.	15 m
					Reduction of pulmonary venous confluence pressure from 21 to 7 mmHg	

*y* years, *m* months, *NR* not rapported, *PAPVC* partially anomalous pulmonary venous connection, *HLHS* hypoplastic left heart syndrome, *TAPVC* total anomalous pulmonary venous connection

In this paper, we present two cases as a feasibility study evaluating the effects of intraoperative balloon dilatation in two adult patients undergoing surgical treatment of cor triatriatum. Based on these experiences and previously reported cases, recommendations are made to guide patient selection for percutaneous treatment of cor triatriatum.

## Case presentation

### Case 1

**Fig. 1:**
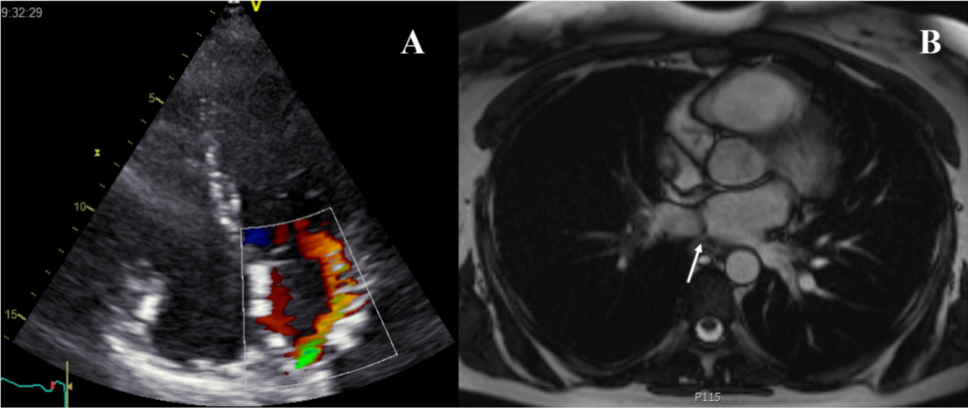
Transthoracic apical 4-chamber view with color Doppler (**a**) showing turbulence in the roof of the left atrium near the right pulmonary vein suggesting pulmonary venous obstruction or stenosis. On cardiac magnetic resonance imaging (**b**), a membrane (*arrow*) was demonstrated in the left atrium, with the left pulmonary veins draining into the left atrium, and the right pulmonary veins into the accessory (proximal) atrial chamber

A 39-year-old woman with an unremarkable medical history was referred to our center with cor triatriatum. The patient was evaluated by her referring cardiologist due to complaints of progressive exertional dyspnea for the past year. Transthoracic and transesophageal echocardiography demonstrated normal left and right ventricular size and function, and a thin membrane in the left atrium dividing the left atrium into 2 compartments, with a mean gradient of 12 mmHg over the membrane. In addition, turbulence was noted in the roof of the left atrium near the right pulmonary vein (PV) suggesting pulmonary venous obstruction or stenosis (Fig. 1a). Cardiac magnetic resonance imaging (MRI) confirmed these findings, and showed that the left pulmonary veins drained in to the left atrium, while the right PVs drained into the accessory (proximal) atrial chamber (Fig. 1b) (type IIIA1 cor triatriatum [3]). Left and right cardiac catheterization revealed normal coronary arteries without significant stenoses, and a significant gradient (10–13 mmHg) between simultaneously measured right pulmonary capillary wedge and left ventricular end diastolic pressure.

**Fig. 2:**
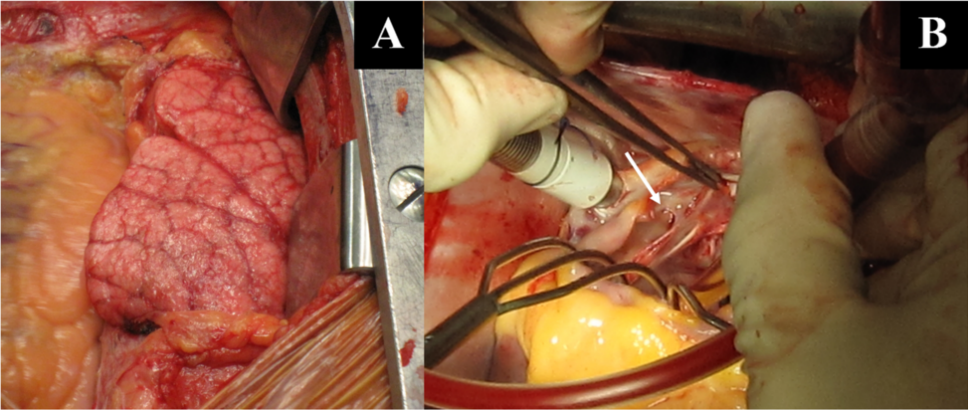
Intraoperatively, marked pulmonary venous congestion of the right lung was found (**a**). After entering the left atrium, a common drainage of the right pulmonary veins was noted, with an obstructing membrane right before the draining orifice, leaving an opening of only 3 mm (**b**, *arrow*)

The patient was operated through median sternotomy, with cardiopulmonary bypass, systemic hypothermia and cardioplegic arrest. There was marked pulmonary venous congestion of the right lung (Fig. 2a). Through a transatrial approach, the left atrium was entered. There was a common drainage of the right PVs, with an obstructing membrane right before the draining orifice, leaving an opening of only 3 mm (Fig. 2b). Intraoperatively, a balloon dilatation was attempted with a 20 mm balloon (2 atm). After several attempts, the orifice diameter was increased to 10 mm, which was deemed insufficient to ensure adequate pulmonary venous drainage of the right side. Complete resection of the membrane was then performed, with surgical enlargement of the right common PV ostium towards the atrial septum to an orifice opening of 20 mm. Reseptation of the atrial septum was performed with xenopericardium to prevent restenosis of the right PV ostium.

Postoperative course was uneventful, and the patient was discharged on postoperative day 7. At 4 months follow-up, the patient was asymptomatic. Echocardiography showed no residual membrane and no signs of pulmonary venous stenosis.

### Case 2

A 58-year-old woman with a history of paroxysmal atrial fibrillation was referred to our center with cor triatriatum. Transthoracic and transesophageal echocardiography demonstrated normal left and right ventricular size and function, and an enlarged left atrium. A thin membrane was noticed in the left atrium dividing it into 2 compartments, with a mean gradient of 4 mmHg over the membrane, which increased to 7 mmHg during exercise. Pulmonary hypertension was noticed (36 mmHg), which increased after exercise (56 mmHg). Cardiac MRI confirmed these findings, demonstrating a proximal chamber receiving all PVs and communicating with the left atrium (type IA [3]). Left and right cardiac catheterization showed normal coronary arteries without significant stenoses, and a significant mean gradient between simultaneously measured right pulmonary capillary wedge and left ventricular end diastolic pressure of 6 mmHg, which increased to 20 mmHg after dopamine-infusion.

**Fig. 3:**
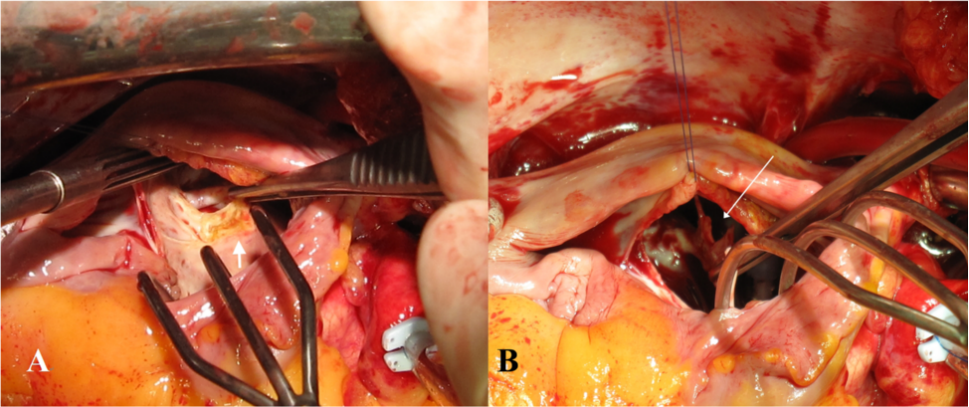
Significant calcification of the inferior border of a cor triatriatum membrane (**a**, *short thick arrow*). After intraoperative balloon dilatation, a tear of 3 mm in the membrane was created next to the calcification (**b**, *long thin arrow*)

Intraoperatively, an obstructing membrane was found between the proximal and distal chamber, with calcification of the inferior border of the membrane and an orifice of 1 cm (Fig. 3a). A balloon dilatation was attempted with a 24 mm balloon (2–3 atm). This created a tear of 3 mm in the membrane next to the calcification (Fig. 3b), and was expanded to 5 mm after an additional dilation. The opening orifice was evidently larger after dilatation, and no extracardiac extension of the tear was noted. Subsequently, the whole membrane (3 × 5 cm) was surgically resected. In addition, bilateral PV isolation was performed (Medtronic Cardioblate irrigated radiofrequency System) with creation of a roofline and trigone line, and excision of the left atrial appendage.

Postoperative course was uneventful, and the patient was discharged on postoperative day 10. At 6 months follow-up, the patient was asymptomatic, with less dyspnea on exertion when compared to before the operation. On echocardiography, no residual membrane was found.

## Conclusions

In these two cases assessing the efficacy of cathether-based interventional strategies for cor triatriatum in the adult, several cautionary lessons can be learned regarding patient selection. While the concept of percutaneous therapy for this congenital abnormality is sound and technically certainly feasible, several anatomical considerations should be taken into account. Various variations exist of cor triatriatum, depending on the drainage of the PVs and the drainage of the proximal chamber in the right or left atrium [3] (Table 1). Only isolated forms of cor triatriatum where all PVs ultimately drain into the left atrium can be recommended for percutaneous strategies. As such, only type IA (classic cor triatriatum where an accessory atrial chamber receives all PVs and connects with the left atrium) and type IIIA1 (subtotal cor triatriatum with an accessory atrial chamber receiving part of the PVs and communicates with the left atrium, with the remaining PVs connecting normally) are amendable for percutaneous intervention. In addition, approximately 75 % of patients in large surgical series undergoing operative treatment also underwent additional surgical procedures [4, 5, 14]. When cor triatriatum is associated with other cardiac abnormalities requiring surgical intervention, operative therapy naturally remains the treatment of choice. Therefore, detailed imaging studies are essential in guiding patient selection for percutaneous therapy, to clarify the exact anatomy of the anomaly and to detect associated cardiac abnormalities. In this regard, transthoracic and transesophageal echocardiography are usually the initial choice of imaging modality. Additionally, cardiac MRI can even more clearly depict the precise morphology of cor triatriatum, demonstrating the relationship between the fibromuscular membrane and the PVs, determining the hemodynamic significance of the obstruction, and detecting associated cardiac abnormalities [15].

In patients with isolated type IA or type IIIA1 cor triatriatum, we speculate that several anatomical characteristics should be evaluated to predict the chance of a successful percutaneous intervention. The exact anatomy of the PVs draining in the proximal chamber should be assessed, with emphasis on the presence of PV obstruction or stenosis, especially at the level of the draining orifice. As illustrated in case 1, the complex anatomy of an obstructing membrane just before an already stenotic draining orifice of the PV, affects the technical success of a balloon dilatation greatly. Although treatment of PV stenosis with angioplasty and stenting has been reported, long-term outcomes are poor with a high degree of restenosis [16, 17]. As such, we do not recommend an attempt at percutaneous intervention for patients with cor triatiatum with concomitant PV stenosis. In these cases, a surgical resection of the obstructive membrane should be performed with surgical enlargement of the PV orifice.

Besides the presence of PV stenosis, we hypothesize that calcification of the obstructing membrane is an additional important aspect to consider. With the increasing diagnostic accuracy of echocardiography, cor triatriatum might be diagnosed more frequently and at an older age [18], when calcification of the obstructing membrane will be more prevalent. As illustrated in case 2 where the inferior border of the membrane was significantly calcified, balloon dilatation led only to a small tear next to the calcified part of the membrane with limited increase of orifice area. In the setting of PMC for mitral stenosis, valve calcification has been associated with poor immediate and long-term results [19] and is a relative contraindication for PMC [6, 7]. Whether membrane calcification is also associated with procedural success in the percutaneous treatment of cor triatriatum remains to be clarified.

Our initial concern regarding extracardiac rupture, one of the main reasons to initiate this feasibility study through a hybrid approach, seems unfounded based on the intraoperative estimation of tissue tear after balloon dilatation. Correspondingly, cardiac rupture after PMC is a rare complication, and is usually puncture-related rather than a direct consequence of balloon dilatation [20].

Even in cases of cor triatriatum with favourable anatomical characteristics where percutaneous interventional is feasible, the long-term patency remains unclear, with 15 months as the longest reported follow-up period (Table 2). In addition, we assume a publication bias towards reporting successful cases is present. Moreover, surgical repair of cor triatriatum, consisting of total resection of the membrane, is usually a simple and straightforward procedure. Although the use of extracorporeal circulation and cardioplegic arrest of the heart is needed, it can be performed with minimal mortality and morbidity. In the largest surgical series available [4] with 66 patients, no mortality was noted in cases operated after 1970, and no surgical reintervention was needed after a median follow-up of 5.4 years. These results should serve as a benchmark for comparison for any other intervention.

Then what is the role of cathether-based interventional strategies for cor triatriatum? In selected patients who are acutely presented with heart failure and are at that time unfit for surgery, percutaneous interventions could be important as a bridge to definite operative therapy [10], or even as definitive treatment [13], both in pediatric [10] and adult cases [13]. Additionally, when cor triatriatum is diagnosed during pregnancy, a percutaneous strategy might be useful when the anatomical variant is compatible, as cardiac surgery and cardiopulmonary bypass increase maternal and fetal risks. In elective cases of isolated cor triatriatum with favorable anatomical characteristics, the option of a percutaneous intervention can be discussed with the patient as an experimental alternative, or in the setting of a comparative study. These cases should be centralized in high-volume percutaneous intervention centers, preferably with extensive experience in PMC, and should be reported in an international registry for research and educational purposes.

In conclusion, the concept of percutaneous cathether-based interventional treatment strategies for cor triatriatum in the adult is sound and should be technically feasible. However, several anatomical characteristics should be considered to predict technical success, such as the location of the membrane, the degree of PV stenosis, the extent of calcification, and the presence of other (congenital) cardiovascular abnormalities. Furthermore, long-term efficacy of this therapy remains to be confirmed. As such, surgical treatment of cor triatriatum remains the mainstay of treatment in adult patients, especially when other cardiovascular anomalies are present which require surgical correction.

## Consent

Written informed consent was obtained from both patients for publication of this Case report and any accompanying images. A copy of the written consent is available for review by the Editor of this journal.

## References

[1] Niwayama G (1960). Cor triatriatum. Am Heart J.

[2] Jorgensen CR, Ferlic RM, Varco RL, Lillehei CW, Eliot RS (1967). Cor triatriatum. Review of the surgical aspects with a follow-up report on the first patient successfully treated with surgery. Circulation.

[3] Herlong JR, Jaggers JJ, Ungerleider RM (2000). Congenital Heart Surgery Nomenclature and Database Project: pulmonary venous anomalies. Ann Thorac Surg.

[4] Yaroglu Kazanci S, Emani S, McElhinney DB (2012). Outcome after repair of cor triatriatum. Am J Cardiol.

[5] Saxena P, Burkhart HM, Schaff HV, Daly R, Joyce LD, Dearani JA (2014). Surgical repair of cor triatriatum sinister: the Mayo Clinic 50-year experience. Ann Thorac Surg.

[6] Nishimura RA, Otto CM, Bonow RO, Carabello BA, Erwin JP, Guyton RA (2014). 2014 AHA/ACC Guideline for the Management of Patients with Valvular Heart Disease: a report of the American College of Cardiology/American Heart Association Task Force on Practice Guidelines. J Am Coll Cardiol.

[7] Vahanian A, Alfieri O, Andreotti F, Antunes MJ, Joint Task Force on the Management of Valvular Heart Disease of the European Society of Cardiology (ESC), European Association for Cardio-Thoracic Surgery (EACTS) (2012). Guidelines on the management of valvular heart disease (version 2012). Eur Heart J.

[8] Kerkar P, Vora A, Kulkarni H, Narula D, Goyal V, Dalvi B (1996). Percutaneous balloon dilatation of cor triatriatum sinister. Am Heart J.

[9] Huang TC, Lee CL, Lin CC, Tseng CJ, Hsieh KS (2002). Use of Inoue balloon dilatation method for treatment of Cor triatriatum stenosis in a child. Catheter Cardiovasc Interv.

[10] Schiller O, Burns KM, Sinha P, Cummings SD (2012). Cor triatriatum with partial anomalous pulmonary venous return: a rare case of parallel obstruction and successful staged treatment. Pediatr Cardiol.

[11] Schranz D, Jux C, Akintuerk H (2013). Novel catheter-interventional strategy for intracardiac connecting of total anomalous pulmonary venous return in newborns with hypoplastic left heart-syndrome prior to hybrid approach. Catheter Cardiovasc Interv.

[12] Sivakumar K, Satish R, Tailor K, Coelho R (2008). Transcatheter management of subtotal cor triatriatum sinister: a rare anomaly. Pediatr Cardiol.

[13] Méndez AB, Colchero T, Garcia-Picart J, Vila M, Subirana MT, Sionis A (2013). Unusual case of new-onset heart failure due to cor triatriatum sinister. Eur J Heart Fail.

[14] Alphonso N, Nørgaard MA, Newcomb A, d’Udekem Y, Brizard CP, Cochrane A (2005). Cor triatriatum: presentation, diagnosis and long-term surgical results. Ann Thorac Surg.

[15] Elagha AA, Fuisz AR, Weissman G (2012). Cardiac magnetic resonance imaging can clearly depict the morphology and determine the significance of cor triatriatum. Circulation.

[16] Abadir S, Sarquella-Brugada G, Mivelaz Y, Dahdah N, Miró J (2009). Advances in paediatric interventional cardiology since 2000. Arch Cardiovasc Dis.

[17] Balasubramanian S, Marshall AC, Gauvreau K, Peng LF, Nugent AW, Lock JE (2012). Outcomes after stent implantation for the treatment of congenital and postoperative pulmonary vein stenosis in children. Circ Cardiovasc Interv.

[18] Ohlow MA, von Korn H, Haberl K, Wagner A, Secknus MA, Yu J (2005). Cor triatriatum sinister in a 61-year-old patient. Cardiology.

[19] Bouleti C, Iung B, Himbert D, Messika-Zeitoun D, Brochet E, Garbarz E (2014). Relationship between valve calcification and long-term results of percutaneous mitral commissurotomy for rheumatic mitral stenosis. Circ Cardiovasc Interv.

[20] Varma PK, Theodore S, Neema PK, Ramachandran P, Sivadasanpillai H, Nair KK (2005). Emergency surgery after percutaneous transmitral commissurotomy: operative versus echocardiographic findings, mechanisms of complications, and outcomes. J Thorac Cardiovasc Surg.

